# Preparation and thermal properties of mineral-supported polyethylene glycol as form-stable composite phase change materials (CPCMs) used in asphalt pavements

**DOI:** 10.1038/s41598-017-17224-1

**Published:** 2017-12-05

**Authors:** Jiao Jin, Feipeng Lin, Ruohua Liu, Ting Xiao, Jianlong Zheng, Guoping Qian, Hongfu Liu, Pihua Wen

**Affiliations:** 1 School of Traffic and Transportation Engineering, Changsha University of Science and Technology, Changsha, 410114 China; 2 Key Laboratory of Special Environment Road Engineering of Hunan Province, Changsha University of Science and Technology, Changsha, 410114 China; 3 School of Minerals Processing and Bioengineering, Central South University, Changsha, 410083 China; 4 School of Engineering and Materials Science, Queen Mary, University of London, London, E1 4NS UK

## Abstract

Three kinds of mineral-supported polyethylene glycol (PEG) as form-stable composite phase change materials (CPCMs) were prepared to choose the most suitable CPCMs in asphalt pavements for the problems of asphalt pavements rutting diseases and urban heat islands. The microstructure and chemical structure of CPCMs were characterized by SEM, FT-IR and XRD. Thermal properties of the CPCMs were determined by TG and DSC. The maximum PEG absorption of diatomite (DI), expanded perlite (EP) and expanded vermiculite (EVM) could reach 72%, 67% and 73.6%, respectively. The melting temperatures and latent heat of CPCMs are in the range of 52–55 °C and 100–115 J/g, respectively. The results show that PEG/EP has the best thermal and chemical stability after 100 times of heating-cooling process. Moreover, crystallization fraction results show that PEG/EP has slightly higher latent heats than that of PEG/DI and PEG/EVM. Temperature-adjusting asphalt mixture was prepared by substituting the fine aggregates with PEG/EP CPCMs. The upper surface maximum temperature difference of temperature-adjusting asphalt mixture reaches about 7.0 °C in laboratory, and the surface peak temperature reduces up to 4.3 °C in the field experiment during a typical summer day, indicating a great potential application for regulating pavement temperature field and alleviating the urban heat islands.

## Introduction

Asphalt pavements have been widely used and account for approximately 90% of highways, which play an important role in road construction due to the unique advantages^1^. However, the color of asphalt pavements is black, and the solar albedo for asphalt pavements ranges from 0.04 to 0.06^2^, while the black asphalt absorbs highly solar radiation and presents a considerably high surface temperature around 70–80 °C during peak sunlight conditions^3^. As a kind of temperature-sensitivity material, the surface temperature has significant effect on the mechanical properties and life span of asphalt pavements. For instance, asphalt pavements become softer when the surface temperature is higher than the softening point of asphalt during summer day, thus leading to permanent deformation under vehicle load, which affects the driving comfort and safety seriously. Moreover, numbers of studies showed that asphalt pavements in cities cover a very large part of the urban fabric, which is quite important to the development of the urban heat island (UHI)^4,5^. Thus, researches should be organized around technologies about reducing the surface temperature of asphalt pavements and mitigating the heat island.

Phase change materials (PCMs) as the key of latent thermal energy storage has attracted interests in different solar-thermal and building energy efficiency area owing to its advantages of high storage density and stable thermal performance^6,7^. Phase change material is a substance with a specific melting point and high heat of fusion, when reaches the temperature of phase change (e.g., melts), it absorbs a great deal of heats at an almost isothermal condition. Therefore, many research efforts have been committed to the preparation of form-stable CPCMs in building materials so that the PCMs can remain structure stable during phase changing period in recent years^8–12^. The utilization of CPCMs could significantly reduce the maximum room temperature during the day and the heating load at night^13^. Therefore, use of PCMs in the mass of the pavements were explored both theoretically and experimentally^14^, which will decrease the surface peak temperature and the amount of sensible heat released to the atmosphere due to higher thermal capacitance of the materials. The asphalt pavements based on PCMs could be a potential choice of adjusting the surface temperature and mitigating urban heat island effects.

Three common methods have been demonstrated for incorporation of PCMs in asphalt pavements: direct incorporation^15^, using pipes of PCMs^16^ and incorporation of form-stable CPCMs in pavements^17–20^. On one hand, the direct incorporation of PCMs (a variety of saturated acids and unsaturated acids) in asphalt may increase the saturated content of asphalt, which will reduce the consistency of asphalt, resulting in higher penetration and lower ductility. On the other hand, in the case where metal tubes filled with PCMs were used, the PCMs were concentrated in a few parts of the pavement and did not provide protection to the entire structure, heat could not able to flow from the PCMs and metal pipes into the pavements because of the low thermal conductivity of asphalt concrete^21^. Furthermore, there are some researches on form-stable CPCMs used in asphalt pavements by sol-gel method in recent years, which process of preparation is cumbersome. Considering all mentioned above, further efforts are required to find a reasonable method of incorporation of PCMs in asphalt mixtures.

In this study, three kinds of mineral-supported CPCMs were prepared to choose the most suitable form-stable CPCMs used in asphalt pavements. The chemical compatibility of prepared CPCMs were characterized by SEM, XRD and FT-IR analysis techniques. The prepared CPCMs were investigated in terms of thermal properties and thermal reliability using DSC and TG analysis techniques. Thermal storage/release performance of CPCMs was conducted. According to the thermal analysis and comparison, the PEG/EP was selected as the most suitable material used in asphalt mixture. Moreover, thermal performance of temperature-adjusting asphalt pavements with PEG/EP was also studied in laboratory and field.

## Experimental section

### Results

#### Morphological analysis

**Figure 1:**
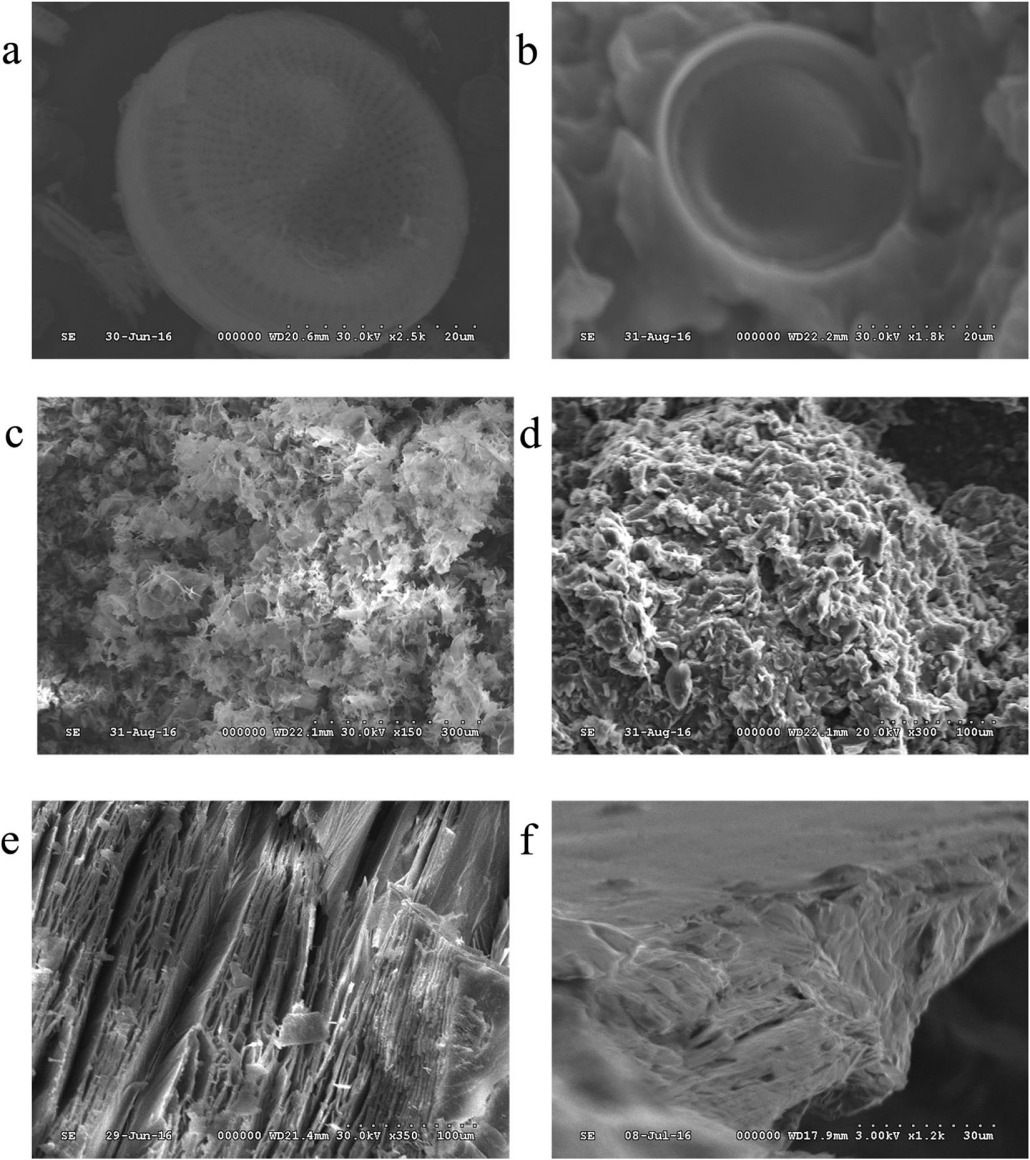
Morphologies of the samples. SEM images of (**a**) DI, (**b**) PEG/DI, (**c**) EP, (**d**) PEG/EP, (**e**) EVM and (**f**) PEG/EVM.

The SEM images of minerals before and after PEG impregnation are shown in Fig. 1. As the carrier, raw DI exhibits highly porous disk-like shape (Fig. 1(a)), numerous pores indicates the high porosity and large specific surface area of DI as expected. PEG is absorbed in DI uniformly, and the primary porous structure morphology of DI as well as interface between PEG and DI are not seen in Fig. 1(b). Figure 1(c) shows that EP is flocculent, these pores vary in size, and the edge is obvious. PEG has been absorbed in the pores and on the surface of EP, and the edge of EP turns smooth and round (Fig. 1(d)). As seen from Fig. 1(e), EVM has irregular layers and uneven pores in the layers. The EVM have rough and random microstructures. The image in Fig. 1(f) of the PEG/EVM also shows that the porous network of EVM was fully filled with PEG. It can be seen that the PEG is completely incorporated into the pores of DI, EP and EVM used as the supporting materials from the SEM images. This porous structure of three minerals provided mechanical durability for the composites and prevented the leakage of the melted PEG due to the consequence of capillary and surface tension force^10,22^.

#### Characterization

**Figure 2:**
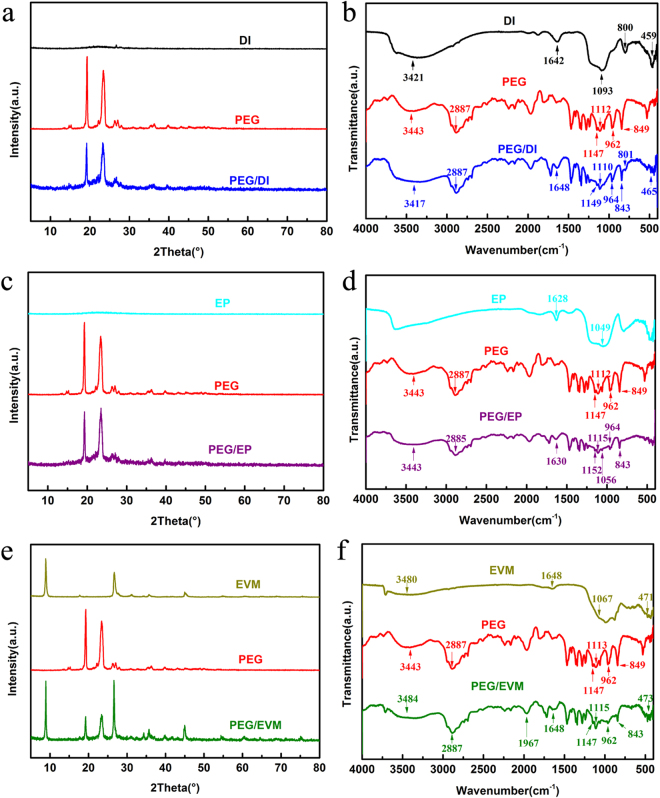
Crystallization and spectra of the samples. (**a**,**b**) PEG/DI, (**c**,**d**) PEG/EP and (**e**,**f**) PEG/EVM.

The XRD pattern of DI, EP, EVM, PEG and PEG/DI, PEG/EP, PEG/EVM composites are shown in Fig. 2(a,c,e). The peak between 20° and 30° represents a typical non-crystalline structure of DI and EP (a, c), and the characteristic peaks of EVM appear at 8.9°, 26.7° and 45° (e). As porous materials, the crystal structure of DI, EP and EVM had not been affected after the incorporation of PEG. Both the characteristic peaks of supports and PEG appear in the XRD patterns of the CPCMs. The FT-IR specta in Fig. 2 (b,d,f) show most of characteristic peaks of the pure PEG. The triplet peak of the C-O-C stretching vibration at 1112 cm^−1^ and 1147 cm^−1^ could be clearly observed. The peaks at 2887 cm^−1^, 962 cm^−1^ and 849 cm^−1^ originate from the strenching vibration of the -CH_2_ functional group, the crystal peak of PEG and C-C-O bonds. The peak at 3443 cm^−1^ is attributed to the stretching vibration of the O-H functional group^10,23^. As seen from Fig. 2(b), the peak at 459 cm^−1^ represents the bending vibration of Si-O. The peak at 800 cm^−1^ results from the vibration of SiO-H group. The peak around 1093 cm^−1^ belongs to the stretching vibration of siloxane (-Si-O-Si-) group. Besides, the peaks at 1642 and 3421 cm^−1^ are designated to the stretching vibration and the bending vibration of single bond -OH functional group, respectively^10,24^. As also seen from Fig. 2(d), the peak near 1636 cm^−1^ is because of the bending vibration of the water used in crystallization. The peak at 1049 cm^−1^ is the characteristic absorption peak of the telescopic vibration of Si-O-Si^25^. Moreover, in the spectrum of EVM (Fig. 2f), the peaks appear at 3480 cm^−1^and 1648 cm^−1^are attributed to -OH stretching and bending vibration of the interlayer water, respectively. The characteristic peaks of Si-O stretching vibration and Si-O-Si bending vibration appear at 1067 cm^−1^ and 471 cm^−1^ respectively. The XRD pattern and FT-IR spectra of the composite PCMs contain both the characteristic peaks of the mineral supports and PEG, no significant new peaks were observed in the XRD pattern or FT-IR spectra of the CPCMs, indicating no chemical reactions between the supports and PEG, the bending features between supports and PEG were mainly caused by hydrogen bonding and physical interaction. All the results showed that PEG has been impregnated into the DI, EP and EVM successfully. Thus, the crystallization states of PEG/DI, PEG/EP and PEG/EVM composites are well preserved and stable.

#### Thermo-gravimetric analysis

**Figure 3:**
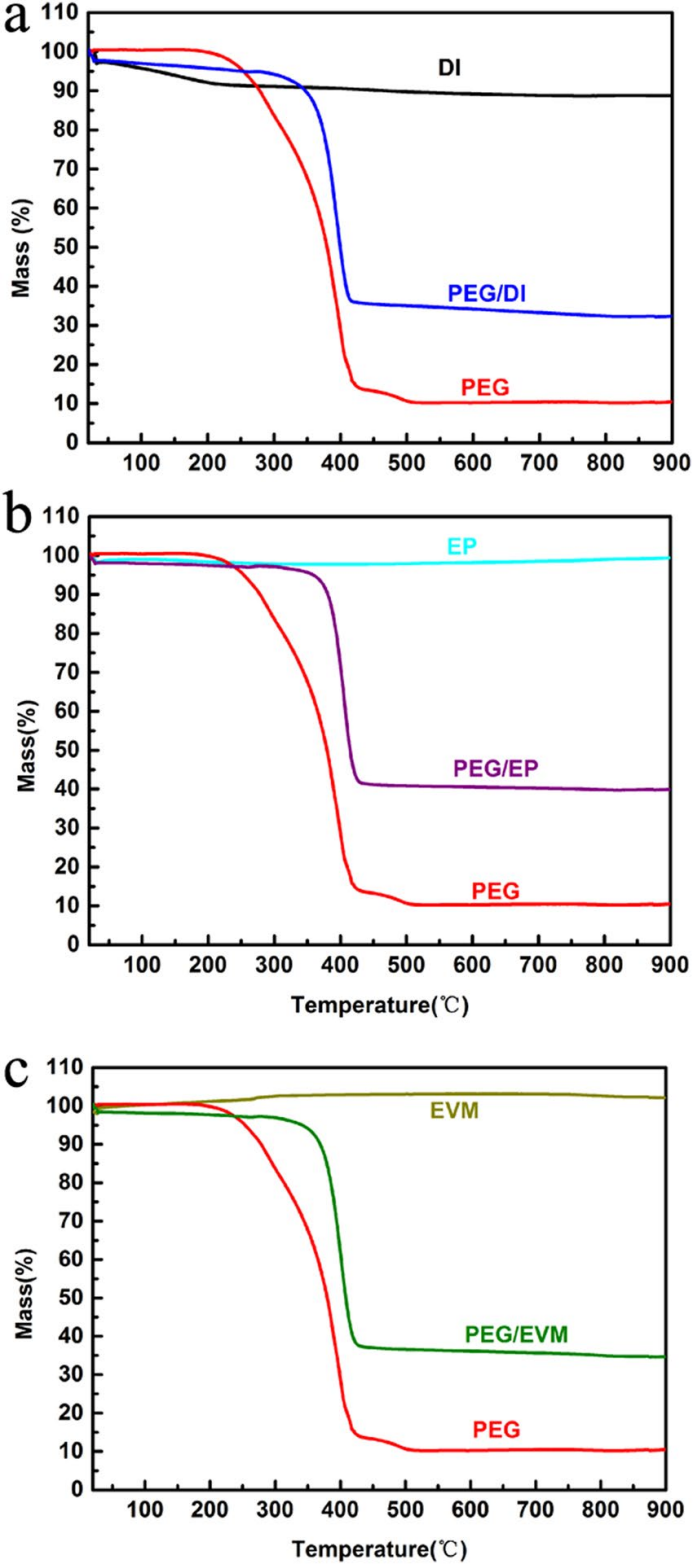
Thermo-gravimetric analysis of the samples. (**a**) PEG/DI, (**b**) PEG/EP and (**c**) PEG/EVM.

Figure 3 shows three thermo-gravimetric curves of PEG, different minerals and the CPCMs. There are no obvious weight loss and decomposition reaction in the range of 25 °C to 200 °C. It shows the mineral-supported PEG as form-stable CPCMs can be used repeatedly below 200 °C. The sharp weight loss in the range of 200 °C to 400 °C is due to PEG decomposition. While the construction temperature of asphalt pavements is usually below 180 °C, the thermal reliability of CPCMs used in asphalt pavements can be retained. As seen from Fig. 3(a), The weight losses for the DI, PEG and PEG/DI below 900 °C were 11.2%, 89.5% and 67.6%, respectively, which included the water steaming of supports and PEG decomposition. It can be concluded that the maximum mass fraction of PEG absorbed into the DI was 72% (assuming the mass fraction of PEG to be x, 11.2%( 1− x) + 89.5%x = 67.6%). Therefore, the mass fraction of PEG absorbed into the EP and EVM were 67% and 73.6% using the similar calculating method from Fig. 3(b,c).

#### Thermal capacities

**Figure 4:**
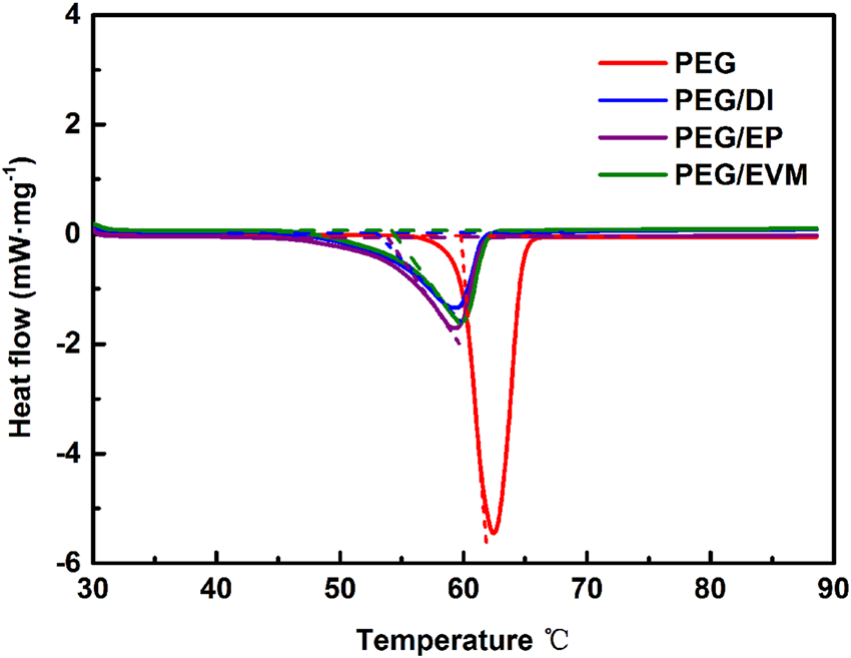
Thermal capacities of the samples. The DSC curves of PEG and the composites.

Thermal capacities (melting temperature and latent heats) of PEG and the CPCMs are determined by the DSC thermal analysis (Fig. 4). Thermal properties of PEG, PEG/DI, PEG/EP and PEG/EVM are given in Table 1. The curve of pure PEG shows a melting temperature (T_m_) at 59.85 °C in the endothermic curve, which is close to the softening point of asphalt. The latent heats of melting (ΔH_m_) pure PEG is 200.7 J/g. The latent heat is a decisive factor for CPCMs and indicates their thermal capacity. The melting latent heats of the composites declined according to the DSC curves of the CPCMs compared with pure PEG. The melting latent heats are determined as 101.9 J/g for PEG/DI, 114.7 J/g for PEG/EP and 111.0 J/g for the PEG/EVM, which are less than their theoretic values (for the PEG/DI, ΔH_m_: 200.7 × 72% = 144.5 J/g, for the PEG/EP, ΔH_m_: 200.7 × 67% = 134.5 J/g, for the PEG/EVM, ΔH_m_: 200.7 × 73.6% = 147.7 J/g). The decrease of the latent heats of the CPCMs not only ascribes to the lower fraction of PEG alone, but also the interactions between PEG and supporting materials, which hampers PEG crystallizing and reduces the latent heats of the CPCMs^26^. The crystallization fraction of the PEG (*F*_*c*_) is calculated by:

$${F}_{c}=\frac{{\rm{\Delta }}{H}_{composite}}{{\rm{\Delta }}{H}_{pure}\beta }$$

where ΔH_composite_ and ΔH_pure_ are the latent heats of the CPCMs and pure PEG, respectively, and β represents the mass fraction of PEG in the composites. As shown in Table 1, the crystallinity of the PEG in PEG/DI and PEG/EVM are 70.5% and 75.2%, respectively, while that in PEG/EP is 85.3% and is larger than that of the other composites, which indicates that PEG/EP has slightly higher latent heats than that of PEG/DI and PEG/EVM even has lower loadage.

**Table 1:** Thermal properties of the PEG and as-synthesized composites.

Sample	Mass fraction of PEG β(%)	Melting Temperature, T_m_ (°C)	Measured latent heat of melting, ΔH_m_ (J/g)	Theoretic latent heat of melting, ΔH_t_ (J/g)	Crystallinity of PEG, F_c_ (%)
PEG	100	59.85	200.7	200.7	100
PEG/DI	72.0	52.91	101.9	144.5	70.5
PEG/EP	67.0	53.86	114.7	134.5	85.3
PEG/EVM	73.6	54.32	111.0	147.7	75.2

#### Thermal storage and release properties

**Figure 5:**
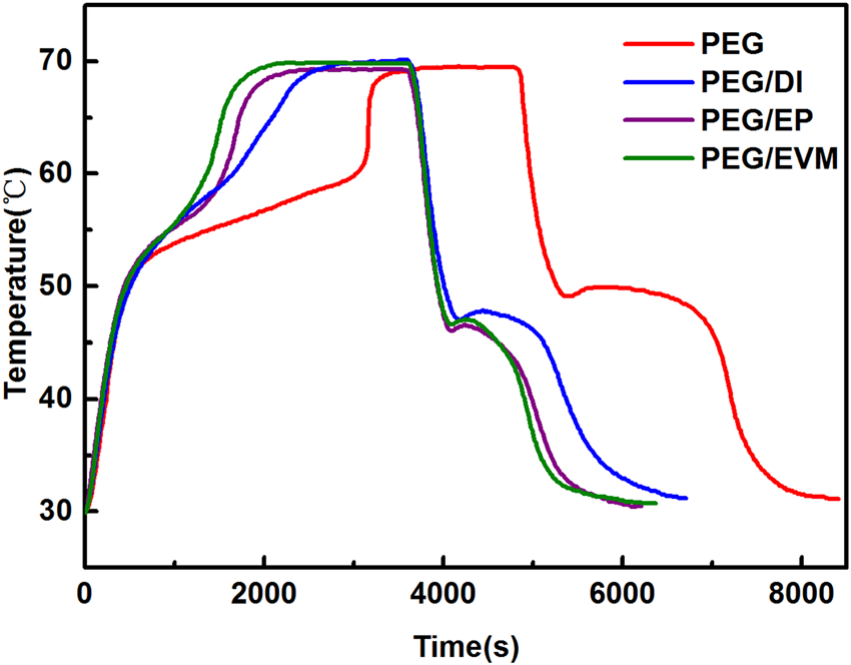
Thermal storage and release properties of the samples. Thermal storage and release curves of PEG and the composites.

The thermal storage and release properties of composites were evaluated by comparing with that of pure PEG. The thermal storage and release curves of the pure PEG and the composites are shown in Fig. 5. In the process of thermal storage, temperature rising rate of the composites and PEG were slowing down in the range of 50–60 °C. The composites and PEG cannot reach the setup temperature of 75 °C, the PEG/DI, PEG/EP and PEG/EVM take 2990 s, 2350 s and 2129 s to reach the equilibrium temperature of 70 °C, respectively, while PEG take 3630 s to reach the equilibrium temperature. In the process of thermal release, it is obvious that the temperature platforms appeared at the range of 45–50 °C. The results show that the thermal storage and release rate of PEG/DI is lower than that of the other composites, which indicates the best performance for delaying the temperature rising of asphalt pavements.

### Discussion

**Figure 6:**
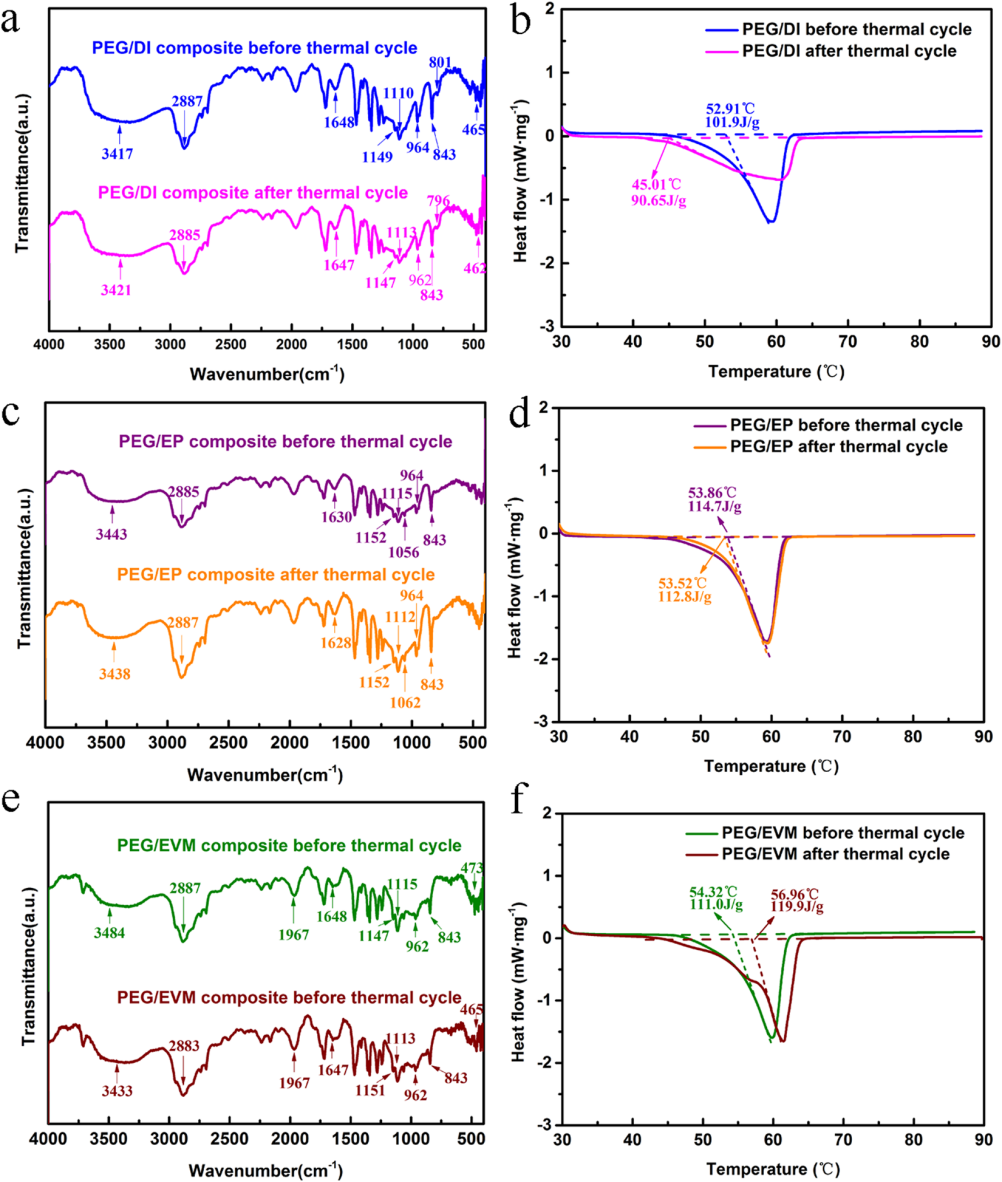
Spectra and thermal capacities of the samples before and after thermal cycling. (**a**,**b**) PEG/DI, (**c**,**d**) PEG/EP and (**e**,**f**) PEG/EVM.

The CPCMs must be chemically and thermally stable, no or less change in its chemical structure and thermal performance after long-term utility period. The change in chemical structure and thermal properties of the composite PCMs was determined by thermal cycling test (100 melting/freezing cycles).The FTIR spectra and DSC curves of composites before and after thermal cycle are shown in Fig. 6. As can be seen from the spectra (Fig. 6a,c and e), the shape and frequency values of all characteristic peaks almost didn’t change after 100 thermal cycle, which demonstrates that no chemical structure was influenced and no reaction happened during thermal cycles. Therefore, the CPCMs are chemically stable after thermal cycle. As also seen from DSC curves (Fig. 6b,d and f), the melting temperature of PEG/DI, PEG/EP and PEG/EVM changed as −7.90 °C, −0.34 °C and 2.64 °C, while the latent heats value of melting changed by −11.0%, −1.7% and 8.0% after the thermal cycling test, respectively. According to the above analysis and comparison, it can be concluded that the CPCMs of PEG/EP has the best thermal reliability with regard to the change in its phase change temperature and latent heats.

**Figure 7:**
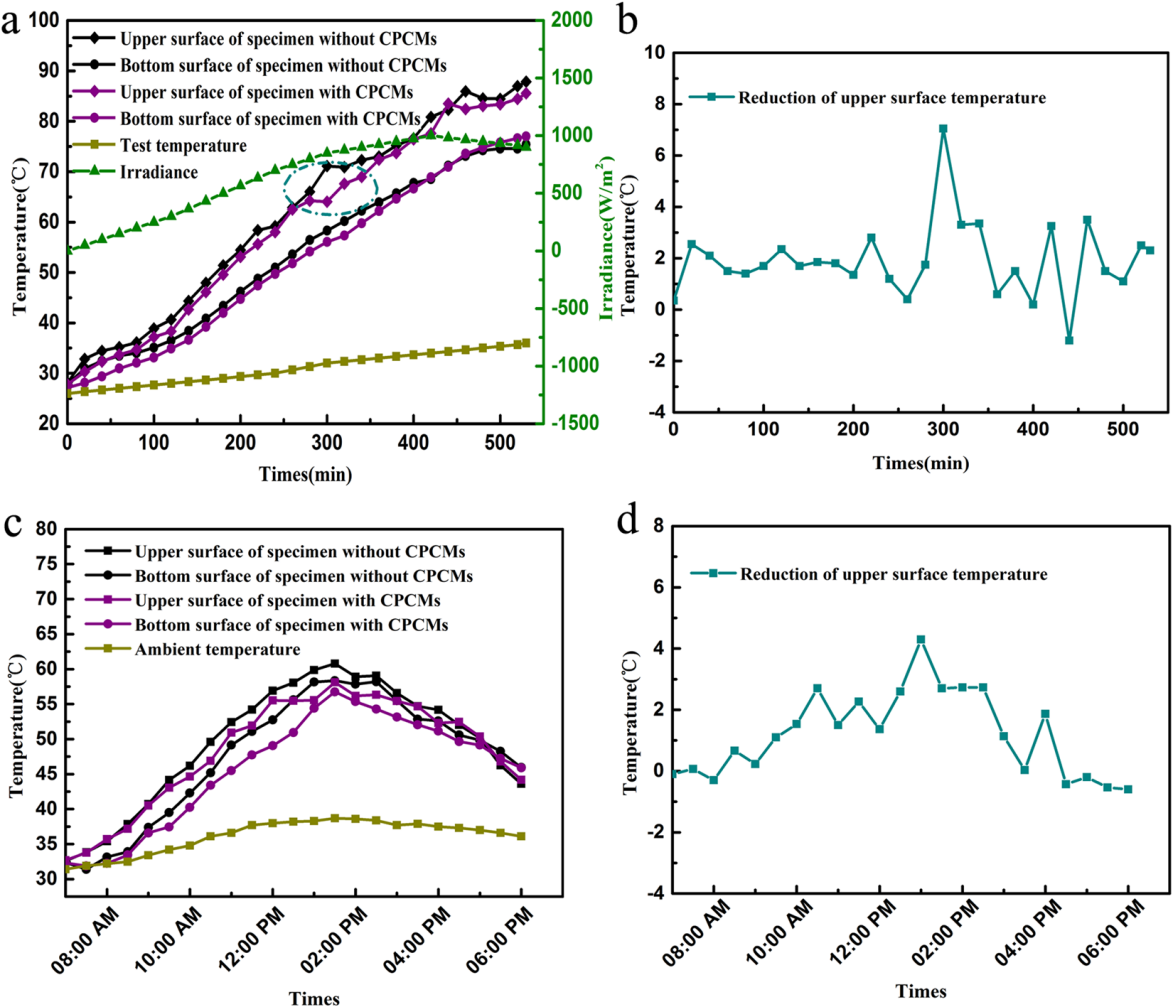
The temperature acquisition. (**a**) the laboratory temperature variations, (**b**) the laboratory temperature reduction, (**c**) the field temperature variations and (**d**) the field temperature reduction.

Figure 7(a) shows the temperature variations at upper and bottom surface of asphalt specimens fabricated with and without CPCMs during heating period in laboratory. The temperature of specimens changes with test temperature and irradiance. And the temperature of upper surface of the former is always lower than that of the latter one. It must be mentioned that there is obvious temperature mitigation at the specimen with CPCMs. Figure 7(b) indicates that the upper temperature difference between two specimens, and the maximum value achieved at 7.0 °C. These results show that the CPCMs in the temperature-adjusting asphalt mixture have been fully phase changed. When time reached at 530 min, the upper surface temperature of two specimens both achieved the maximum, which with CPCMs is 2.3 °C lower than that without CPCMs. The field temperature variations at upper and bottom surface of asphalt specimens are shown in Fig. 7(b,c). The experimental results indicate that the temperature-adjusting asphalt mixture can reduce the surface peak temperature of pavements by up to 4.3 °C during a typical summer day. The field temperature reduction of upper surface is consistent with the observation from the laboratory.

In summary, here we report a new approach for mineral-supported PEG as form-stable CPCMs used in asphalt pavements. The PEG/EP has the best thermal reliability according to the changes in its phase change temperature and latent heats after 100 thermal cycling test. The temperature-adjusting asphalt mixture with PEG/EP could have a great potential application for regulating pavement temperature field and alleviating the urban heat islands.

### Methods

#### Materials

Polyethylene glycol (PEG4000, AR, m.p.: 59.85 °C) was used as component during the preparation of the form-stable CPCMs. The raw diatomite (DI), expanded perlite (EP) and vermiculite was collected from Jilin, Henan and Xinjiang province in China, respectively. The pristine vermiculite was first thermally expanded at 900 °C for 1 h to produce expanded vermiculite (EVM). Three kinds of minerals (DI, EP and EVM) were chosen to support PEG as form-stable CPCMs. All of the minerals were previously grinded and sieved by 300 μm-mesh sieve and then dried at 120 °C for 2 h. As for asphalt mixture, an asphalt SK AH-70 was obtained from Xiamen Huate Ltd., Co. in Fujian province, China, with penetration of 67 (0.1 mm at 25 °C, 100 g and 5 s), ductility of more than 100 cm at 15 °C, and softening point of 47.5 °C. The Aggregates were obtained by crushed basalt mineral with maximal size of 13 mm.

#### Preparation

The form-stable CPCMs were using vacuum impregnation method^27–29^. PEG and different minerals were placed inside a flask, the magnetic stirrer was turned on, which extracted in thermostatical water bath for 30 min at 90 °C allow PEG to cover the minerals. Then, the vacuum pump was also turned on. After 60 min of vacuum process, air was allowed to enter the flask again to force the liquid PEG to penetrate into the pore space of different minerals. After cooling, three kinds of CPCMs (PEG/DI, PEG/EP, PEG/EVM) was thermally filtered to remove superfluous PEG at 90 °C for 24 h.

#### Characterization

The surface microstructure and morphology of minerals and CPCMs were investigated by scanning electron microscope (SEM), S-3000N + EX-250 model. Fourier transformation infrared (FT-IR) spectra of the samples were measured using KBr as disperse phase in the range of 4000–400 cm^−1^ by a Nicolet NEXUS 670 FTIR spectroscopy. X-ray diffraction (XRD) patterns were performed on Rigaku D/max 2550 diffractometer with Cu K_α_ radiation (λ = 0.15406 nm) over a scanning range of 2*θ* = 5–80° with a step width of 0.02°, and at a voltage of 40 kV and a current of 200 mA. Thermal properties of form-stable CPCMs, such as melting temperature and latent heats, were measured by differential scanning calorimetry (DSC) analysis. The DSC (TA instruments, Q10) measurements were carried out with constant heating rate of 5 °C/min, in the temperature range of 30–90 °C under a constant stream of nitrogen at atmospheric pressure. The melting temperature was taken as onset temperature point of the heating peaks in DSC measurements. The latent heats of phase change were determined by numerical integration of the area under the peaks. The thermal stability and absorption ratio of PEG in the CPCMs were determined by thermogravimetric (TG) analysis. TG (HCT-1 model) was performed at heating rate of 10 °C/min from room temperature up to 900 °C in a nitrogen atmosphere.

#### Thermal cycling test

Thermal cycling test was performed to determine the thermal reliability of form-stable CPCMs in terms of the change in phase change temperatures and latent heats after thermal cycling. The composites were placed inside sealed beakers at 20 °C and 90 °C for 10 min, respectively. And the test was carried out consecutively up to 100 cycling. FT-IR and DSC analyses were repeated to determine the chemical and thermal stability of the CPCMs after thermal cycling.

#### Thermal storage and release test

To confirm the thermal storage/release performance of the CPCMs, thermal storage and release test was also conducted. The CPCMs were filled into identical round-bottomed flasks separately. One digital thermometer with a precision of ±0.1 °C was placed at the center of each flask for temperature measurement. Firstly, the flasks were put inside in the thermostatical water bath at 30 °C for temperature equilibrium. Later, the flasks were put into the thermostatical water bath at 75 °C immediately. After the temperature of samples reached temperature equilibrium, the flasks were put into the thermostatical water bath at 30 °C again. The temperature variations of samples measured by digital thermometer were recorded per 10 second.

#### Temperature-adjusting asphalt mixture and thermal performance test

The thermal performances of asphalt pavements were tested in laboratory and field to determine the effects of form-stable CPCMs. The full-depth asphalt specimens used in this experiment were 150 mm × 150 mm × 50 mm in dimension. The asphalt concrete surface course was obtained based on Marshall design method. In the gradation of asphalt mixtures, fine aggregates of 0.3 mm and 0.15 mm were substituted with corresponding particle size PEG/EP CPCMs at the replacement levels of 50%. The selected mix gradations are listed in Supplementary Table S1.

The asphalt specimens with and without CPCMs were demoulded and put into the heat preservation box, which specimens were tiled 20 mm thick earth soil and wraped the insulation material around, the schematic diagram of heat preservation box is shown in Figure S1(a,c). Then the box was placed into the device for measuring the thermal properties of pavement materials, which the irradiance and test temperature of the device set as 0–1000 W/m^2^ and 26–36 °C to simulates the thermal environment of a summer day from 5 a.m. to 2 p.m. The schematic diagram of test device is shown in Figure S1(b,d). There are thermocouples used ditto to test and record the temperature changes of upper and bottom surface over time. When the sensor of specimens with and without CPCMs displays both temperature are almost the same, Xenon lamp was turned on and the temperature changes was recorded over time. All the temperature of upper and bottom surface will be calculated the average, respectively. Moreover, the rutting specimens were put at outside so that they could be exposed to direct sunlight at a thermal environment of a summer day from 7 a.m. to 6 p.m. as shown in Figure S2.

## Electronic supplementary material

Supplementary Information
