# Supplementary material for: Preparation and thermal properties of mineral-supported polyethylene glycol as form-stable composite phase change materials (CPCMs) used in asphalt pavements
Source: Sci Rep. 2017 Dec 5;7:16998. doi: 10.1038/s41598-017-17224-1 (PMC5717135; doi:10.1038/s41598-017-17224-1)
Supplement: Supplementary file 1 — Supplementary Information [file 41598_2017_17224_MOESM1_ESM.doc]

**Supplementary Information**

**Preparation and thermal properties of mineral-supported polyethylene glycol as form-stable composite phase change materials (CPCMs) used in asphalt pavements**

Jiao Jin ^1,2,*^, Feipeng Lin ^1,2^, Ruohua Liu ^3,*^, Ting Xiao ^1,2^, Jianlong Zheng ^1,2^, Guoping Qian ^1,2^, Hongfu Liu ^1,2^, Pihua Wen ^1, 4^

^1^ *School of Traffic and Transportation Engineering, Changsha University of Science and Technology, Changsha 410114, China*

^2^ *Key Laboratory of Special Environment Road Engineering of Hunan Province，Changsha University of Science and Technology, Changsha 410114, China*

^3^ *School of Minerals Processing and Bioengineering, Central South University, Changsha 410083, China*

^4^ *School of Engineering and Materials Science,* *Queen Mary, University of London,* *London E1 4NS, UK*

^*^ Corresponding author E-mail: jinjiao@csust.edu.cn; Tel.: +86-731-8525 8575; Fax: +86-731-8525 8375

E-mail: ruohualiu@csu.edu.cn; Tel.: +86-731-8887 9622; Fax: +86-731-8871 0801

**Table S1**  **The selected mix gradations.**

| Sieves (mm) | 16 | 13.2 | 9.5 | 4.75 | 2.36 | 1.18 | 0.6 | 0.3 | 0.15 | 0.075 | Mineral powder | CPCMs |
| --- | --- | --- | --- | --- | --- | --- | --- | --- | --- | --- | --- | --- |
| Passing rate (%) | 100 | 96 | 80 | 47 | 32.5 | 21 | 15 | 11 | 7 | 5 | - | - |
| Component  (%) | - | 4 | 16 | 33 | 14.5 | 11.5 | 6 | 2 | 2 | 2 | 5 | 4 |

**
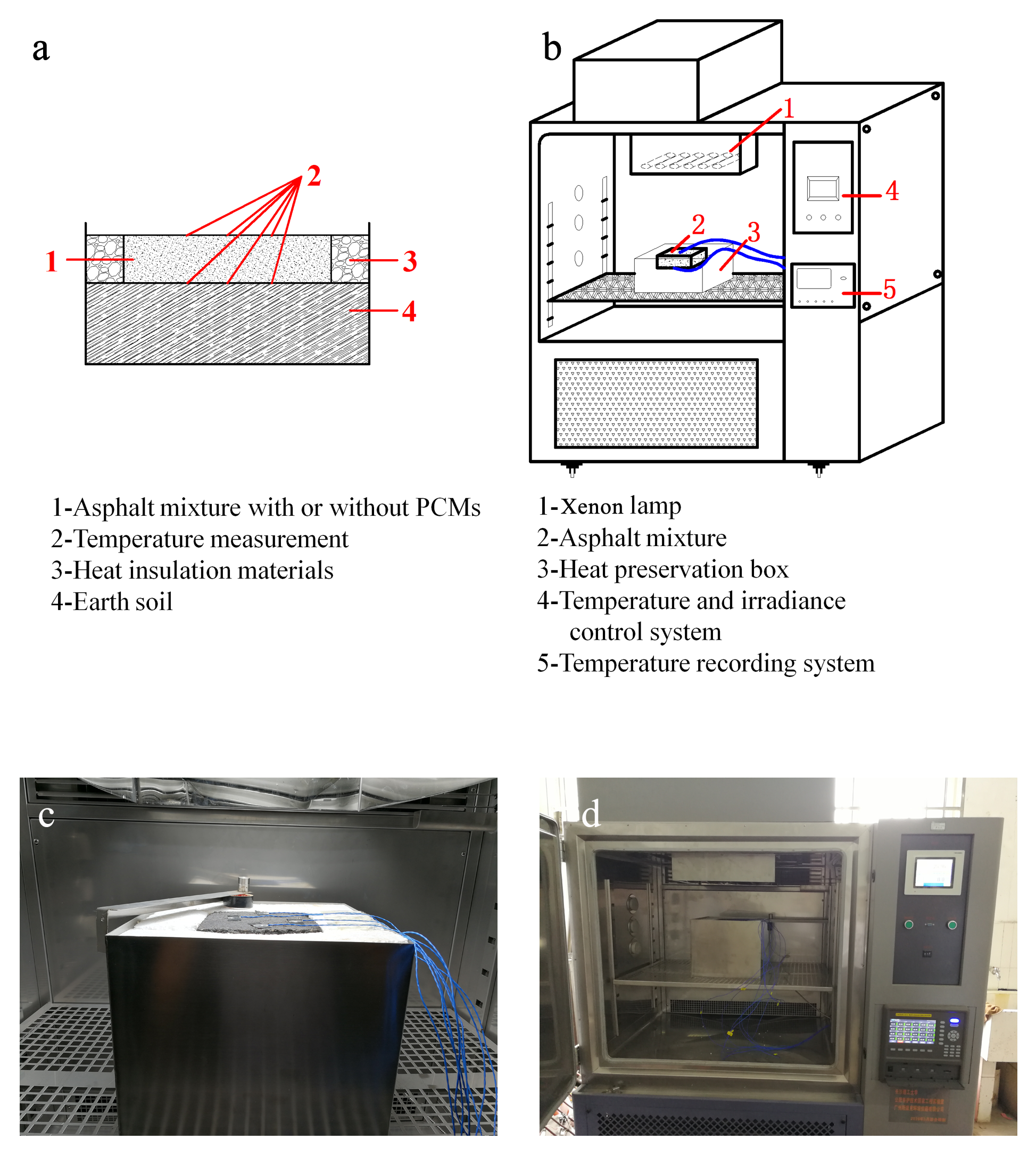
**

**Figure S1**  **The laboratory temperature acquisition:** (a) (c) Heat preservation box; (b) (d) Device for measuring the thermal properties of pavement materials;


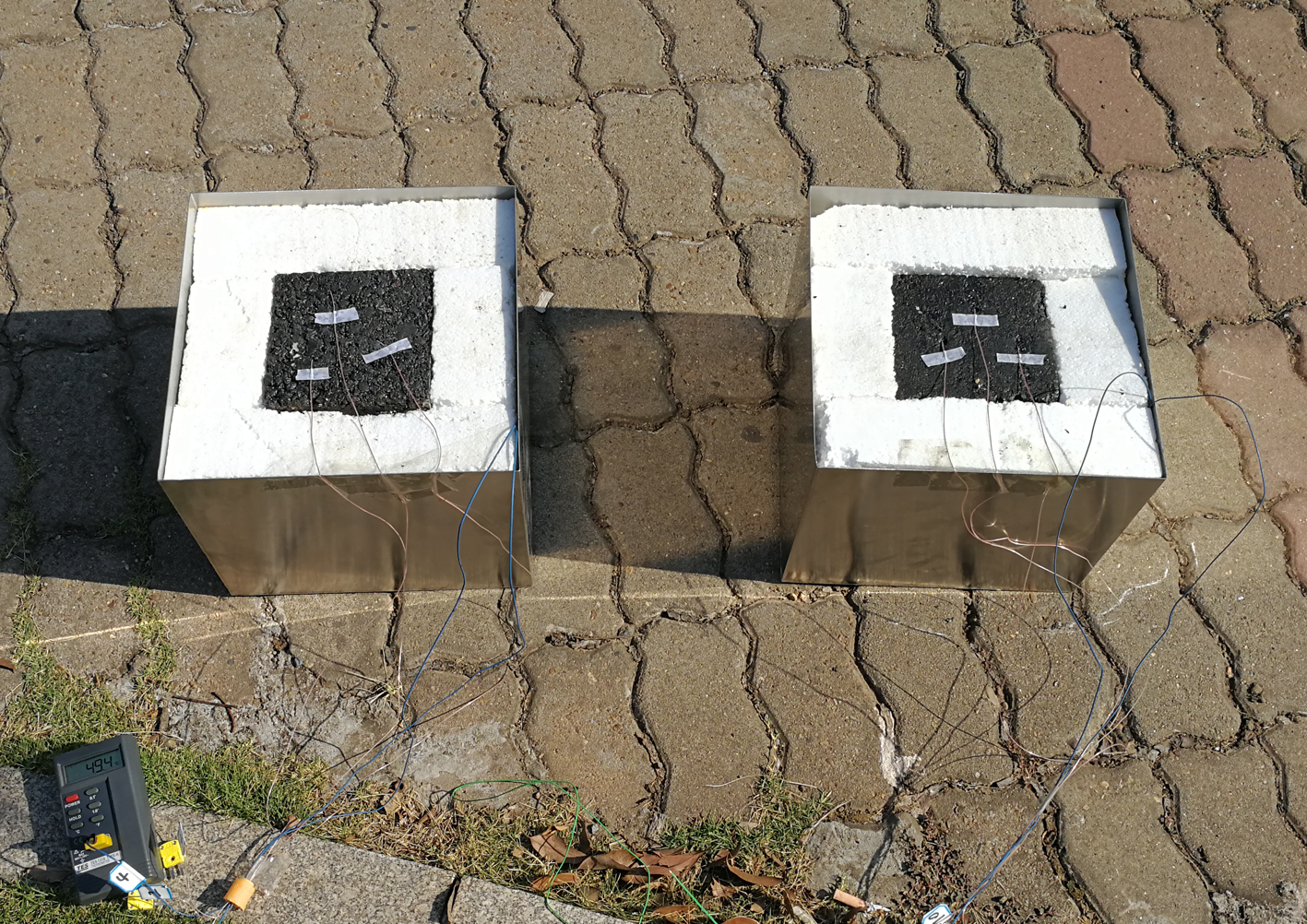


**Figure S2**  **The field temperature acquisition.**
